# Combining all-trans retinoid acid treatment targeting myeloid-derived suppressive cells with cryo-thermal therapy enhances antitumor immunity in breast cancer

**DOI:** 10.3389/fimmu.2022.1016776

**Published:** 2022-11-01

**Authors:** Yue Lou, Peng Peng, Shicheng Wang, Junjun Wang, Peishan Du, Zelu Zhang, Jiamin Zheng, Ping Liu, Lisa X. Xu

**Affiliations:** School of Biomedical Engineering and Med-X Research Institute, Shanghai Jiao Tong University, Shanghai, China

**Keywords:** cryo-thermal therapy, all trans retinoid acid, combination therapy, myeloid-derived suppressive cells, T cell immunity

## Abstract

Targeting myeloid-derived suppressive cells (MDSCs) has been considered a potential strategy in tumor therapy. However, a single drug targeting MDSCs remains a challenge in the clinic. An increasing number of studies have shown that combination agents targeting MDSCs and immunotherapy may provide exciting new insights and avenues to explore in tumor therapy. In our previous study, a novel cryo-thermal therapy was developed for metastatic tumors that systematically activate innate and adaptive immunity. Moreover, cryo-thermal therapy was shown to dramatically decrease the levels of MDSCs and induce their differentiation toward potent antigen-presenting cells. However, the therapeutic effects of cryo-thermal therapy on the 4T1 mouse breast cancer model were still not satisfactory because of the high level of MDSCs before and after treatment. Therefore, in this study, we combined cryo-thermal therapy with all-trans retinoid acid (ATRA), a small molecule drug that can induce the inflammatory differentiation of MDSCs. We found that combination therapy notably upregulated the long-term survival rate of mice. Mechanically, combination therapy promoted the phenotype and functional maturation of MDSCs, efficiently decreasing suppressive molecule expression and inhibiting glutamine and fatty acid metabolism. Moreover, MDSCs at an early stage after combination therapy significantly decreased the proportions of Th2 and Treg subsets, which eventually resulted in Th1-dominant CD4^+^ T-cell differentiation, as well as enhanced cytotoxicity of CD8^+^ T cells and natural killer cells at the late stage. This study suggests a potential therapeutic strategy for combination ATRA treatment targeting MDSCs with cryo-thermal therapy to overcome the resistance of MDSC-induced immunosuppression in the clinic.

## Introduction

T cell-based immunotherapy has shown remarkable clinical efficacy in multiple cancer types; however, the accumulation of immunosuppressive cells limits the therapeutic efficacy (1). Myeloid-derived suppressive cells (MDSCs) are considered to be suppressive and inhibit antitumor immunity to promote tumor progression (2). The level of MDSCs in peripheral circulation or tumor tissue is highly related to poor prognosis (3–5). Thus, strategies to overcome the immune tolerances induced by MDSCs are urgently needed.

MDSCs are a highly heterogeneous group of cells that coexpress CD11b and Gr-1 in tumor-bearing mice and are the direct progenitors of dendritic cells (DCs), macrophages, and granulocytes (6). MDSCs facilitate the differentiation of immature DCs, tumor-associated macrophages, and Tregs by secreting suppressive or chronic cytokines, expressing immune checkpoint molecules, and inducing oxidative stress and nutrient depletion (7). Currently, Food and Drug Administration (FDA)-approved medicines fail to prevent the accumulation and function of MDSCs. However, combining antitumor therapies targeting MDSCs with other therapies improves the prognosis of patients (8), which may provide a new strategy for tumor therapy.

In our previous studies, a novel tumor cryo-thermal therapy was established to entirely ablate primary tumor tissue through alternating liquid nitrogen cooling and radiofrequency (RF) heating (9). Moreover, cryo-thermal therapy remodels the immunosuppressive environment by inducing durable Th1-induced antitumor immune protection and decreasing the levels of effective Tregs (10, 11). Cryo-thermal therapy significantly improved the cure rate in both the B16F10 melanoma model and the 4T1 triple-negative breast cancer model (12–14). The therapeutic effect of cryo-thermal therapy was observed in different tumor models, and approximately 50% of mice exhibited long-term survival in the 4T1 model, while more than 80% of B16F10-bearing mice were cured (12–14). To understand the different mouse survival rates after cryo-thermal therapy in the two models, immune status before treatment was analyzed. The proportion of MDSCs in the B16F10 model was significantly lower than that in the 4T1 model. Moreover, although the proportion of MDSCs was notably reduced after cryo-thermal therapy, the level of MDSCs in the 4T1 model was still higher than that in the B16F10 model. Therefore, MDSCs may be the major barrier to improving the therapeutic efficacy of cryo-thermal therapy in highly immunosuppressive tumor models.

To further improve the therapeutic efficacy of cryo-thermal therapy in the 4T1 tumor model, which has a high level of MDSCs, in this study, we combined all-trans retinoic acid (ATRA) treatment targeting MDSCs with cryo-thermal therapy. ATRA can stimulate the differentiation of MDSCs into mature DCs and macrophages (15). We found that combination therapy could significantly downregulate the suppressive function of MDSCs and prevent the differentiation of suppressive subsets of CD4^+^ T cells at the early phase, which ultimately resulted in Th1-dominant differentiation of CD4^+^ T cells at the late phase and improved the long-term survival rates of 4T1-bearing mice. Our study provides a new therapeutic strategy combining ATRA treatment targeting MDSCs with cryo-thermal therapy for highly immunosuppressive tumors.

## Materials and methods

### Cell cultivation and animal model

The 4T1 breast cancer cell line was provided by Shanghai First People’s Hospital, China, and the female BALB/c mice were obtained from Shanghai Slaccas Experimental Animal Co., Ltd. (Shanghai, China). Tumors were established by subcutaneous injection of 4T1 cells (4 × 10^5^ cells in 100 μl of phosphate-buffered saline (PBS)) in the right flank of wild-type BALB/c mice as above (11). All animal experiments were approved by the Animal Welfare Committee of Shanghai Jiao Tong University, and experimental methods were performed in accordance with the guidelines of Shanghai Jiao Tong University Animal Care (approved by Shanghai Jiao Tong University Scientific Ethics Committee, Proto code 2020017).

### Cryo-thermal therapy and all-trans retinoid acid treatment

The cryo-thermal therapy system was developed and maintained by Dr. Aili Zhang and Engineer Jincheng Zou in our laboratory. When the diameter of the tumor reached 10 mm (about 16 days after tumor inoculation), mice were randomly divided into tumor-bearing control or cryo-thermal group. Mice from the cryo-thermal group were anesthetized with 5% chloral hydrate (Sinopharm Chemical Reagent Co., Ltd., Shanghai, China) and treated with fast liquid N_2_ freezing at −20°C for 5 min followed by RF heating at 50°C for 10 min to entirely ablate the primary tumor tissue. The parameter of cryo-thermal therapy was determined according to our previous studies. The maximum power was set as 65 W, and the real-time power was adjusted according to temperature.

For ATRA treatment, 200 μg of ATRA (dissolved in corn oil) was administered daily by i.p. from the day before cryo-thermal therapy to 3 days after treatment. The single cryo-thermal therapy group was treated with the same dose of corn oil.

### Flow cytometry analysis

The splenocytes were separated into single-cell suspension, and the red blood cells were removed by erythrocyte-lysing reagent (containing 0.15 M of NH_4_Cl, 1.0 M of KHCO_3_, and 0.1 mM of Na_2_EDTA). The dead cells were excluded by Zombie Dye staining, and then cells were stained by a cell-specific surface marker for 20 min. For transcription factors detection, cells were treated with a True-Nuclear Transcription Factor Buffer Set (BioLegend, San Diego, CA, USA) according to the manufacturer’s protocol. For intercellular staining, cells were stimulated with Cell Activation Cocktail (with Brefeldin A, 20 µg/ml, BioLegend, San Diego, CA, USA) for 4 h before Zombie Dye and surface marker staining. After that, cells were fixed and permeabilized with the fixation buffer and permeabilization wash buffer according to the manufacturer’s instructions (BioLegend, San Diego, CA, USA) and followed by intercellular cytokines staining for 20 min. For data collection, BD FACS Aria II cytometer (BD Biosciences, Franklin Lakes, NJ, USA) was used, and the data were analyzed using FlowJo software (https://www.flowjo.com accessed on 3 July 2022). All the fluorochrome-conjugated monoclonal antibodies were purchased from BioLegend (San Diego, CA, USA) and listed in **Supplementary Table 1**.

### RNA isolation and qRT-PCR

Total RNA was isolated using TRIzol Reagent (TaKaRa, Otsu, Shiga, Japan) and was reverse transcribed to cDNA by the PrimeScript RT reagent kit (TaKaRa, Otsu, Shiga, Japan). The absorbance at 260/280 nm of samples above 1.9 was used. Quantitative real-time PCR (qRT-PCR) was performed on the ABI 7900HT sequence detection system, and SDS 2.4 software (Applied Biosystems, Waltham, MA, USA) was used for Ct value collection. The expression level of targeted genes was normalized as GAPDH (ΔΔCt method).

### Isolation of CD4^+^ T cells and myeloid-derived suppressive cells

For isolation of CD4^+^ T cells, single-cell suspensions of splenocytes from 4T1 tumor-bearing control mice (day 16 after inoculation) were isolated by an Easysep CD4^+^ T cell negative selection kit (StemCell Technologies, Vancouver, BC, Canada). For isolation of MDSCs, single-cell suspensions of splenocytes from indicated groups were firstly incubated with Gr1-PE (listed in **Supplementary Table 1**) and then isolated with a PE positive selection kit (StemCell Technologies, Vancouver, BC, Canada).

### *In vitro* coculture assay

For detection of CD4^+^ T-cell differentiation influenced by MDSCs, splenic MDSCs separated from the indicated group of mice were cocultured with the CD4^+^ T cells isolated from the spleen of tumor-bearing mice before treatment (16 days after tumor inoculation) in the ratio of 1:5 for 24 h. Corresponding serums were added to mimic the cytokine environment *in vivo*.

For detection of the suppressive function of MDSCs on the proliferation of CD4^+^ T cells, splenocytes from age-paired naïve mice were labeled with carboxyfluorescein succinimidyl ester (CFSE) and then cocultured with MDSCs in the indicated E/T ratio with anti-CD3 (1 ng/ml, BioLegend, San Diego, CA, USA) added to stimulate the proliferation of T cells. After 72 h, the proliferated CD4^+^ T cells (CFSE^−^) were tested by flow cytometry.

### Gene expression profiling

RNA samples from freshly isolated splenic MDSCs from tumor-bearing control, cryo-thermal therapy, or combination therapy were extracted using the mirVana miRNA Isolation Kit (Ambion, Austin, TX, USA) and constructed into libraries using TruSeq Stranded mRNA LTSample Prep Kit (Illumina, San Diego, CA, USA) following the manufacturer’s protocol. Then the libraries were sequenced on the Illumina sequencing platform (HiSeq™ 2500), and 125 bp/150 bp paired-end reads were generated. The fold change of gene expression >2.0 was considered significantly different. The principal component analysis (PCA) was performed using OECloud tools (https://cloud.oebiotech.cn accessed on 3 July 2022). The gene set enrichment analysis was performed using GSEA as described (16). The gene set LIT_MM_LAROSA_FATTY-ACID-BIOSYNTHESIS_METABOLISM_DIFF was used for fatty acid metabolism analysis (p-value <0.05).

### Statistical analysis

Student’s t-test with a two-tailed distribution was used for statistical comparisons using Graph Pad Prism 7 (https://www.graphpad.com accessed on 3 July 2022). Figures denote statistical significance of *p < 0.05, **p < 0.01, ***p < 0.001, and ****p < 0.0001. p-values <0.05 were considered to be statistically significant.

## Results

### Cryo-thermal therapy facilitated the maturation and differentiation of myeloid-derived suppressive cells and induced their suppressive phenotype

**Figure 1:**
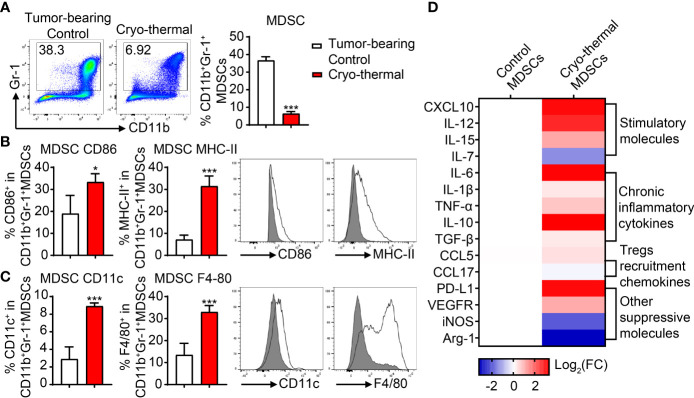
The proportion and phenotype of MDSCs after cryo-thermal therapy. **(A)** The proportion of splenic MDSCs on day 21 after cryo-thermal therapy. **(B, C)** The expression levels of CD86 and MHC-II **(B)**, CD11c, and F4/80 **(C)** on MDSCs from tumor-bearing control (gray) and cryo-thermal therapy (solid line) were detected by flow cytometry. **(D)** The expression levels of functional molecules of MDSC were detected by qRT-PCR. *p < 0.05, ***p < 0.001. n = 4 for each group. MDSCs, myeloid-derived suppressive cells.

In our previous study, we found that in the 4T1 model, the proportion of MDSCs in the spleen of mice was decreased within 14 days and was then slightly increased on day 21 after cryo-thermal therapy [14]. Because high levels of MDSCs contribute to tumor progression, we supposed that the increased level of MDSCs on day 21 after cryo-thermal therapy would affect the therapeutic effect of cryo-thermal therapy. To identify changes in the differentiation and proliferation of MDSCs after cryo-thermal therapy, 4T1-bearing mice were treated with cryo-thermal therapy, and on day 21 after treatment, the proportion of splenic MDSCs (marked as CD11b^+^Gr-1^+^) was examined by flow cytometry. As shown in **Figure 1A**, the proportion of MDSCs was notably decreased after cryo-thermal therapy compared with that in tumor-bearing mice. Moreover, the expression levels of CD86 and MHC-II were significantly upregulated, which indicated the phenotypic maturation of MDSCs (**Figure 1B**), and the expression levels of CD11c and F4/80 were markedly increased, which suggested the differentiation of MDSCs into functional inflammatory DCs and macrophages (**Figure 1C**). To further clarify the function of MDSCs, splenic MDSCs were separated by magnetic beads, and the expression levels of stimulatory and suppressive molecules were detected by qRT-PCR. Compared to those of tumor-bearing mice, the expression levels of stimulatory molecules, including CXCL10, IL-12, and IL-15, were significantly upregulated, while the expression level of IL-7 was downregulated (**Figure 1D**). However, the expression levels of chronic inflammatory cytokines (IL-6, IL-1β, TNF-α, IL-10, TGF-β, etc.), which contribute to the immunosuppressive function of MDSCs, and other suppressive molecules (PD-L1, VEGFR, etc.), as well as the chemokine that participates in Treg recruitment (CCL5), were increased in MDSCs after cryo-thermal therapy (**Figure 1D**). However, the expression levels of iNOS and Arg-1 were downregulated after cryo-thermal therapy (**Figure 1D**). These results suggested that immunostimulatory and suppressive markers of MDSCs were both increased after cryo-thermal therapy.

### Myeloid-derived suppressive cells promoted the proliferation of CD4^+^ T cells while inhibiting Th1 differentiation and increasing the proportion of Th2 cells and Tregs after cryo-thermal therapy

**Figure 2:**
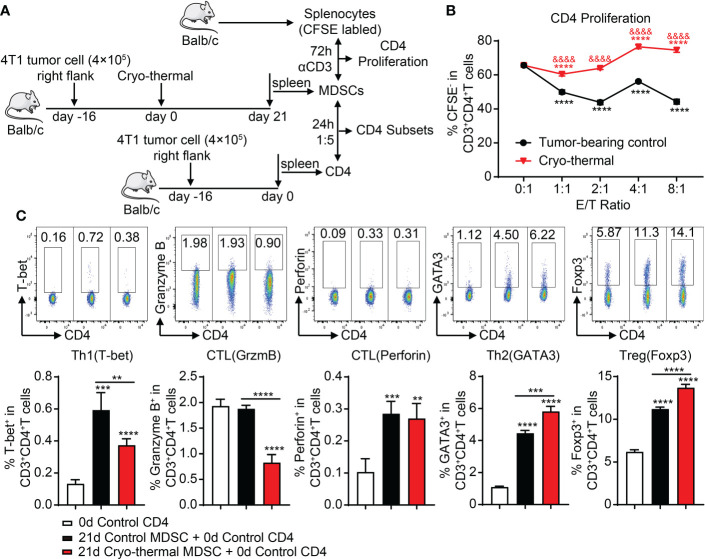
MDSCs after cryo-thermal therapy facilitate the proliferation of CD4^+^ T cells but also the immunosuppressive subset differentiation. **(A)** Scheme of study design. In brief, MDSCs from the spleen of tumor-bearing mice or cryo-thermal treated mice (day 21 after treatment) were separated by MACS. To detect the function of MDSCs on CD4^+^ T-cell proliferation, MDSCs were cocultured with the splenocytes from age-paired naïve mice in the indicated E/T ratio with anti-CD3 (1 ng/ml) added to stimulate the proliferation of T cells. The splenocytes were pre-labeled with CFSE. After 72 h, the proliferated CD4^+^ T cells (CFSE^−^) were tested by flow cytometry. To analyze the function of MDSCs on the differentiation of CD4^+^ T cells, MDSCs were cocultured with the splenic CD4^+^ T cells separated from the tumor-bearing mice before treatment (16 days after tumor inoculation) in the ratio of 1:5. The corresponding serums were added to mimic the cytokine environment *in vivo*. The subsets of CD4^+^ T cells were detected after 24 h by flow cytometry. **(B, C)** The proliferation **(B)** and subsets of CD4^+^ T cells **(C)** after coculture with MDSCs *in vitro*. **p < 0.01, ***p < 0.001, ****p < 0.0001. The symbol & in panel **B** means the comparison with tumor-bearing control, & p < 0.0001. n = 4 for each group. MDSCs, myeloid-derived suppressive cells; MACS, magnetic-activated cell sorting; CFSE, carboxyfluorescein succinimidyl ester.

In our previous studies, we found that Th1-dominant differentiation of CD4^+^ T cells was essential for cryo-thermal-induced antitumor immune response (17). However, MDSCs can directly suppress the proliferation and cytotoxic differentiation of T cells while inducing the differentiation of Tregs (7). Considering that cryo-thermal-induced MDSCs expressed both immunosuppressive and immunostimulatory markers, splenic MDSCs were cocultured with CD4^+^ T cells to investigate the effect of MDSCs on the proliferation and differentiation of CD4^+^ T cells on day 21 after cryo-thermal therapy. The schematic of the study design is shown in **Figure 2A**. Splenic MDSCs from tumor-bearing mice showed a strong capacity to inhibit the proliferation of CD4^+^ T cells, while after cryo-thermal therapy, splenic MDSCs could promote the proliferation of CD4^+^ T cells at a high E/T ratio (**Figure 2B**). However, compared to MDSCs from the tumor-bearing control group, MDSCs in the cryo-thermal therapy group significantly decreased Th1 differentiation and inhibited the expression of granzyme B in CD4^+^ T cells (**Figure 2C**). Moreover, the proportions of Th2 cells and Tregs were increased when cocultured with MDSCs after cryo-thermal therapy compared with those from tumor-bearing mice (**Figure 2C**). These results indicated that after cryo-thermal therapy, the suppressive effect of MDSCs could not be entirely reversed, which mainly inhibited the differentiation of CD4^+^ T cells toward the Th1 subset and promoted CD4^+^ T suppressive subset differentiation.

### Combining all-trans retinoid acid with cryo-thermal therapy decreased the differentiation of CD4^+^ T cells into suppressive subsets at an early stage

**Figure 3:**
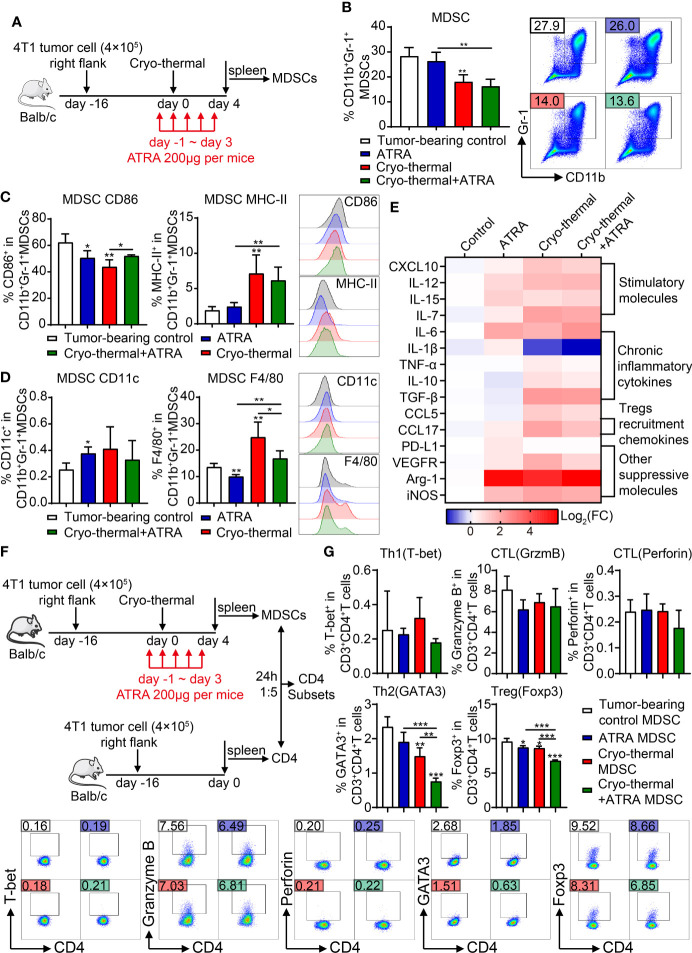
Combination therapy downregulated the suppressive function of MDSCs and promote inflammatory differentiation of CD4^+^ T cells. **(A)** Scheme of study design. In brief, 200 μg of ATRA was administered daily from the day before cryo-thermal therapy to day 3 after treatment, and the phenotype of MDSCs and the subsets of CD4^+^ T cells were detected on day 4 by flow cytometry. **(B–D)** The proportion **(B)**, maturation phenotype **(C)**, and transmission phenotype **(D)** of MDSCs. **(E)** The molecule expression profiles of splenic MDSCs were detected by qRT-PCR on day 4 after treatment. **(F, G)** MDSCs after combination therapy inhibited the differentiation of CD4^+^ T cells into immunosuppressive subsets. **(F)** Scheme of study design. In brief, MDSCs from the spleen of different groups of mice on day 4 after treatment were separated and cocultured with the splenic CD4^+^ T cells separated from the tumor-bearing mice before treatment (16 days after tumor inoculation) in the ratio of 1:5. The corresponding serums were added to mimic the cytokine environment *in vivo*. The subsets of CD4^+^ T cells were detected after 24 h by flow cytometry. **(G)** The subsets of CD4^+^ T cells after coculture with MDSCs *in vitro*. *p < 0.05, **p < 0.01, ***p < 0.001. n = 4 for each group. MDSCs, myeloid-derived suppressive cells; ATRA, all-trans retinoid acid.

As stated previously, the level of MDSCs was decreased after cryo-thermal therapy; however, their suppressive function was not entirely reversed. ATRA can induce the differentiation of MDSCs into mature DCs and macrophages and inhibit the suppressive function of MDSCs. Therefore, we combined ATRA with cryo-thermal therapy (combination therapy) to further convert immunosuppressive MDSCs into immunostimulatory cells. To determine whether combination therapy could trigger the maturation of MDSCs at an early stage after treatment, 200 μg of ATRA was injected by i.p. from the day before cryo-thermal therapy to 3 days after treatment, and the phenotypes of MDSCs were analyzed on day 4 after therapy (**Figure 3A**). As shown in **Figure 3B**, ATRA treatment alone did not decrease the level of MDSCs compared with that in the tumor-bearing control group, while cryo-thermal therapy alone significantly decreased the percentage of MDSCs compared with that in the tumor-bearing control group. However, combination therapy did not further reduce the proportion of MDSCs (**Figure 3B**). To determine whether combination therapy could facilitate the maturation and differentiation of MDSCs, the expression level of surface markers, including CD86, MHC-II, CD11c, and F4/80 on MDSCs, was further examined. ATRA treatment alone downregulated the expression levels of CD86 and F4/80 and slightly increased the level of CD11c on MDSCs compared to those in the tumor-bearing control group, which indicated that ATRA treatment alone could not regulate the phenotypic maturation of MDSCs (**Figures 3C, D**). After cryo-thermal therapy, compared to that in the tumor-bearing control group, the expression level of MHC-II on MDSCs was upregulated, and the level of CD86 was downregulated (**Figures 3C, D**). Interestingly, combination therapy did not affect the expression of MHC-II but increased the expression level of CD86 compared to cryo-thermal therapy, which suggested that cryo-thermal therapy combined with ATRA could further promote the maturation of cryo-thermal-induced MDSCs (**Figures 3C, D**). The expression level of F4/80 on MDSCs in the combination therapy group was increased compared to ATRA treatment alone but was lower than that in the cryo-thermal therapy group, and the level of CD11c on MDSCs after combination therapy was not different compared to that of the other groups (**Figures 3C, D**). These results suggested that combination therapy mainly promoted the inflammatory activity of MDSCs.

To further analyze the signature of MDSCs after combination therapy, the expression levels of functional molecules in MDSCs were detected by qRT-PCR. As shown in **Figure 3E**, compared to those of splenic MDSCs from tumor-bearing mice, ATRA treatment alone slightly increased the expression levels of some inflammatory molecules in MDSCs, such as IL-12, IL-7, and IL-15, but the level of IL-6, which is considered a chronic inflammatory cytokine and plays a major role in MDSC-induced immunosuppression (18), was also increased. Moreover, the expression level of suppressive molecules, including CCL17, PD-L1, VEGFR, Arg-1, and iNOS, was also increased after ATRA treatment alone (**Figure 3E**). These results indicated that after ATRA treatment alone, MDSCs maintained a suppressive signature. Cryo-thermal therapy alone dramatically upregulated the expression profiles of MDSCs compared to tumor-bearing controls, except for the downregulation of IL-1β and an unchanged level of PD-L1. Similar expression profiles of MDSCs were observed after combination therapy; however, the expression levels of TNF-α, IL-1β, CCL5, CCL17, VEGFR, and iNOS were further downregulated after combination therapy compared to cryo-thermal therapy alone. These results indicated that combination therapy could further downregulate the expression of suppressive molecules in MDSCs while retaining the level of inflammatory molecules induced by cryo-thermal therapy.

Therefore, we hypothesized that MDSCs induced after combination therapy would affect the differentiation of CD4^+^ T cells. Then, an *in vitro* coculture experiment was performed. Splenic MDSCs were isolated from the treated mice on day 4 after therapy and sorted for coculture with CD4^+^ T cells from tumor-bearing mice at a ratio of 1:5, and the subsets of CD4^+^ T cells were analyzed after 24 h (**Figure 3F**). Compared to MDSCs from tumor-bearing mice, MDSCs from ATRA-treated mice slightly decreased the percentage of Tregs, and MDSCs from cryo-thermal treated mice slightly decreased the proportion of Th2 and Treg cells, while MDSCs from the combination therapy group further downregulated the proportions of Th2 cells and Tregs (**Figure 3G**). However, MDSCs did not promote the expansion of Th1 cells or increase cytotoxic functions (**Figure 3G**). These results indicated that at the early stage after combination therapy, the differentiation of suppressive CD4^+^ T cell subsets induced by MDSCs was impaired, but Th1 and CTL differentiation were not enhanced.

### Combination therapy induced Th1-dominant CD4^+^ T-cell differentiation at the late stage

**Figure 4:**
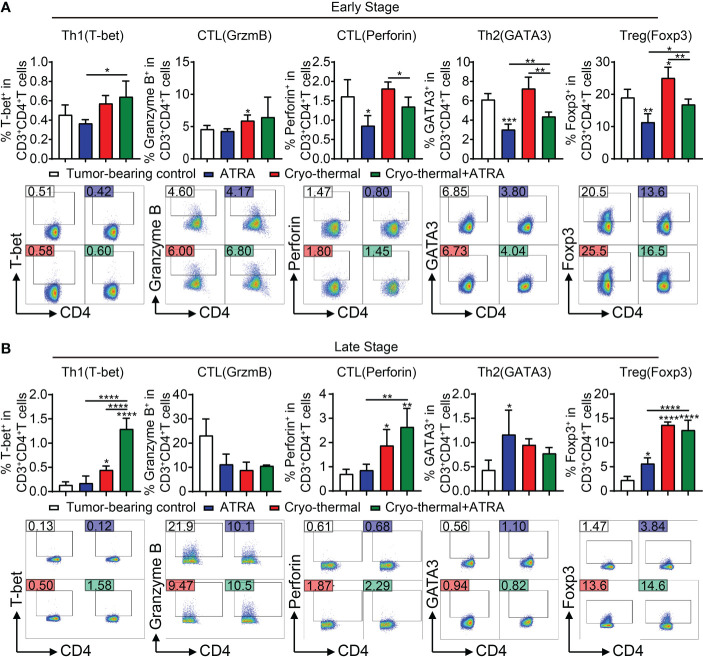
*In vivo* CD4^+^ T-cell differentiation at early and late stages after combination therapy. **(A)** The subsets of CD4^+^ T cells were detected by flow cytometry at an early stage (day 4). **(B)** The subsets of CD4^+^ T cells were detected by flow cytometry at a late stage (day 21). *p < 0.05, **p < 0.01, ***p < 0.001, ****p < 0.0001. n = 4 for each group.

To further determine that combination therapy facilitates the maturation of MDSCs, which decrease the differentiation of suppressive CD4^+^ T subsets, *in vivo* subsets of CD4^+^ T cells at the early stage (4 days after therapy) were analyzed by flow cytometry. Compared to the tumor-bearing control, ATRA treatment alone significantly decreased the differentiation of Th2 cells and Tregs, but the expression level of perforin in CD4^+^ T cells was obviously decreased; furthermore, the Th1 subset showed a decreasing trend (**Figure 4A**). On the contrary, after cryo-thermal therapy, the percentages of CD4-CTLs and Tregs were both increased compared with those in tumor-bearing controls (**Figure 4A**). These suggested that these single therapies could not induce Th1-dominant differentiation of CD4^+^ T cells. However, combination therapy significantly increased the proportion of Th1 subsets compared to ATRA treatment alone and decreased the level of Th2 cells and Tregs compared to cryo-thermal therapy alone (**Figure 4A**), which suggested that combination therapy would have the ability to induce Th1-dominant differentiation of CD4^+^ T cells at the early stage after treatment. Furthermore, the proportion and function of other lymphocytes, including CD8^+^ T cells and NK cells, were also examined. After combination therapy, CD8^+^ T cells showed higher expression levels of IFN-γ, and the expression level of perforin was increased in NK cells than after ATRA treatment alone; however, compared to the effect of cryo-thermal therapy alone, the proportion of CD8^+^ T cells was decreased, and the expression level of perforin was reduced (**Supplementary Figure 1**). These results indicated that combination therapy mainly affects CD4^+^ T-cell-dominant immune response and inhibited suppressive subset differentiation at the early stage.

Although the subsets of Th2 cells and Tregs were notably decreased by combination therapy at the early stage, the increase in Th1 cells and CD4-CTL subsets was not observed compared with the effect of cryo-thermal therapy alone. Therefore, we evaluated CD4^+^ T-cell subsets at the late stage after treatment (21 days after cryo-thermal therapy). Compared to those in the tumor-bearing control, ATRA treatment alone upregulated the levels of Th2 cells and Tregs (**Figure 4B**), which suggested that ATRA treatment alone could not enhance the CD4^+^ T-cell-mediated antitumor immune response but could induce immunosuppression. In the cryo-thermal therapy group, the differentiation of Th1 and CTL subsets was markedly promoted, but the level of Tregs was also increased (**Figure 4B**). After combination therapy, the differentiation of Th1 CD4^+^ T cells was further increased but did not change the percentages of CTLs or Th2 cells compared to cryo-thermal therapy alone (**Figure 4B**). However, after combination therapy, the proportion of Tregs maintained the same high level as compared to cryo-thermal alone. Recent studies reveal that combinatorial immunotherapies can exert an antitumor effect by promoting Treg fragility, which characterizes as impaired suppressive function and enhancing partial molecule expression of the Th1 subset, such as high expression of IFN-γ and T-bet (19). In our previous study, we also found that cryo-thermal therapy could drive the fragility of Tregs, and these Th1-like Tregs downregulated the expression of inhibitory molecules (11). After combination therapy, higher levels of T-bet and IFN-γ in Tregs were observed (**Supplementary Figure 2**). These results suggested that combination therapy decreased CD4^+^ T suppressive subsets at an early stage and promoted the Th1 antitumor immunity induced by cryo-thermal therapy at a late stage. Moreover, the proportions of CD8^+^ T cells and NK cells were notably increased after combination therapy compared to ATRA treatment or cryo-thermal therapy alone, and the cytotoxicity of CD8^+^ T cells and NK cells was also enhanced, with the increased expression level of IFN-γ in CD8^+^ T cells, as well as granzyme B, perforin, and IFN-γ in NK cells (**Supplementary Figure 3**). These results suggested that combination therapy could not only induce a Th1-dominant CD4^+^ T-cell-mediated antitumor immunity but also comprehensively promote CD8^+^ T-cell activation and enhance the cytotoxicity of NK cells at the late stage.

### Combination therapy converted the transcriptional and metabolic programs of myeloid-derived suppressive cells at the late stage

To determine whether the Th1-dominant differentiation of CD4^+^ T cells at the late stage was attributed to the reprogramming of MDSCs after combination therapy, the phenotype of MDSCs at the late stage after combination therapy (21 days after cryo-thermal therapy) was examined by flow cytometry. The results showed that ATRA treatment alone had little impact on the proportion and phenotype of MDSCs, while cryo-thermal therapy alone significantly decreased the percentage of MDSCs and increased the expression levels of CD86, MHC-II, CD11c, and F4/80 compared to those in the tumor-bearing control (**Supplementary Figure 4**). However, combination therapy showed no significant difference compared to cryo-thermal therapy alone (**Supplementary Figure 4**).

**Figure 5:**
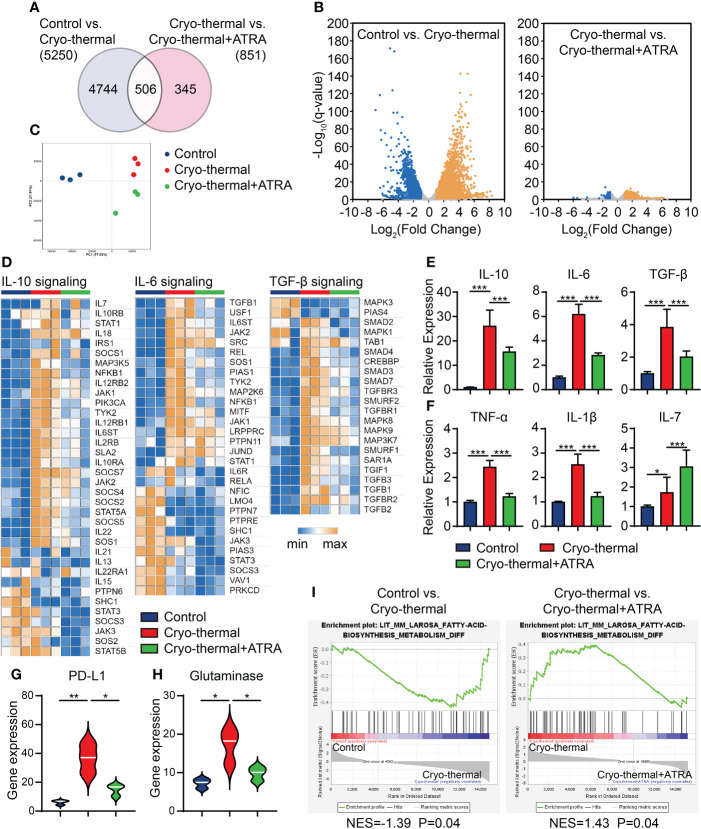
Gene expression profile of MDSCs. Mice were treated with single cryo-thermal therapy or combination therapy, and splenic MDSCs were isolated on day 21 through Gr-1^+^ magnetic bead separation. The gene expression signature of MDSCs was detected by RNA sequence. **(A)** Venn diagram showing the number of statistically significant genes between tumor-bearing control and cryo-thermal therapy, and between cryo-thermal therapy and combination therapy. **(B)** Volcano map of the different gene expressions. **(C)** Principal component analysis of transcriptomes. **(D)** Heatmap of mean fold-change of IL-10, IL-6, and TGF-β-signaling associated gene expression. **(E, F)** The relative expression levels of cytokines in MDSCs were detected by qRT-PCR. **(G, H)** The gene expression of PD-L1 and glutaminase of MDSCs. **(I)** Gene set enrichment analysis of the fatty acid biosynthesis metabolism of MDSCs. *p < 0.05, **p < 0.01, ***p < 0.001. n = 3 for each group. MDSCs, myeloid-derived suppressive cells.

To further understand the role of ATRA in remodeling MDSC function after combination therapy, RNA-seq analysis of splenic MDSCs was performed at the late stage. The gene expression signature of MDSCs showed an enormous difference after cryo-thermal therapy compared with that in tumor-bearing controls, in which the expression levels of 5,250 genes were changed (3,188 upregulated and 2,062 downregulated; **Figures 5A, B**). In addition, compared to those in MDSCs from cryo-thermal therapy and combination therapy, only 851 differentially expressed genes were obtained (706 upregulated and 145 downregulated), indicating a similar immune state after cryo-thermal or combination therapy (**Figures 5A, B**). Among all the different expression genes, 506 genes were changed commonly, when comparing cryo-thermal therapy with tumor-bearing control and comparing combination therapy with cryo-thermal therapy. Among these genes, the changes of most genes were opposite. PCA assessment of all genes showed that samples from two treatment groups were clustered well, and the groups were completely separated. No cross-mixing was found between the two treatment groups, which indicated that the gene expression profiles in these groups were different (**Figure 5C**).

We further studied the expression of suppressive function-related genes in MDSCs, and the IL-10, IL-6, and TGF-β signaling pathways were upregulated after cryo-thermal therapy compared with those in tumor-bearing control. The heatmap analysis showed that after cryo-thermal therapy, these signaling pathways were activated in MDSCs; however, combination therapy significantly inhibited their activation (**Figure 5D**). The qRT-PCR analysis also confirmed that combination therapy could effectively decrease the expression levels of IL-10, IL-6, and TGF-β (**Figure 5E**). In addition, compared to cryo-thermal therapy, combination therapy further reduced the expression levels of other chronic inflammatory cytokines in MDSCs, including TNF-α and IL-1β (**Figure 5F**). Moreover, the expression level of IL-7, a cytokine that is crucial for triggering proliferation, overcoming exhaustion, and improving effector functions of T cells (20), was further upregulated (**Figure 5F**). Furthermore, combination therapy reduced the expression level of PD-L1 on MDSCs (**Figure 5G**). These results suggested that combination therapy could impair the suppressive function of MDSCs through cytokine and checkpoint molecule expression.

Recently, some metabolic pathways of MDSCs have been highlighted for their tumorigenic functions, especially the metabolism of glutamine (21) and fatty acids (22), which have been reported to increase the suppressive functions of MDSCs through multiple pathways. To further evaluate the functional changes in MDSCs after single cryo-thermal and combination therapy, the metabolic diversity of MDSCs was analyzed after the two different therapies. As shown in **Figure 5H**, after cryo-thermal therapy, the expression level of glutaminase, the key enzyme responsible for the transformation of glutamine to glutamate (23), was significantly upregulated compared with that in the tumor-bearing control, but the expression level of glutaminase was downregulated after combination therapy compared to cryo-thermal therapy alone, which suggested that glutamine metabolism was significantly decreased by combination therapy. Additionally, gene set enrichment analysis showed that fatty acid biosynthesis was enriched in the cryo-thermal group but was downregulated after combination therapy (**Figure 5I**). These results indicated that the metabolism of MDSCs could be entirely reversed after combination therapy, which could directly attenuate the suppressive function of MDSCs.

### Combination therapy improved the long-term survival rates of mice

**Figure 6:**
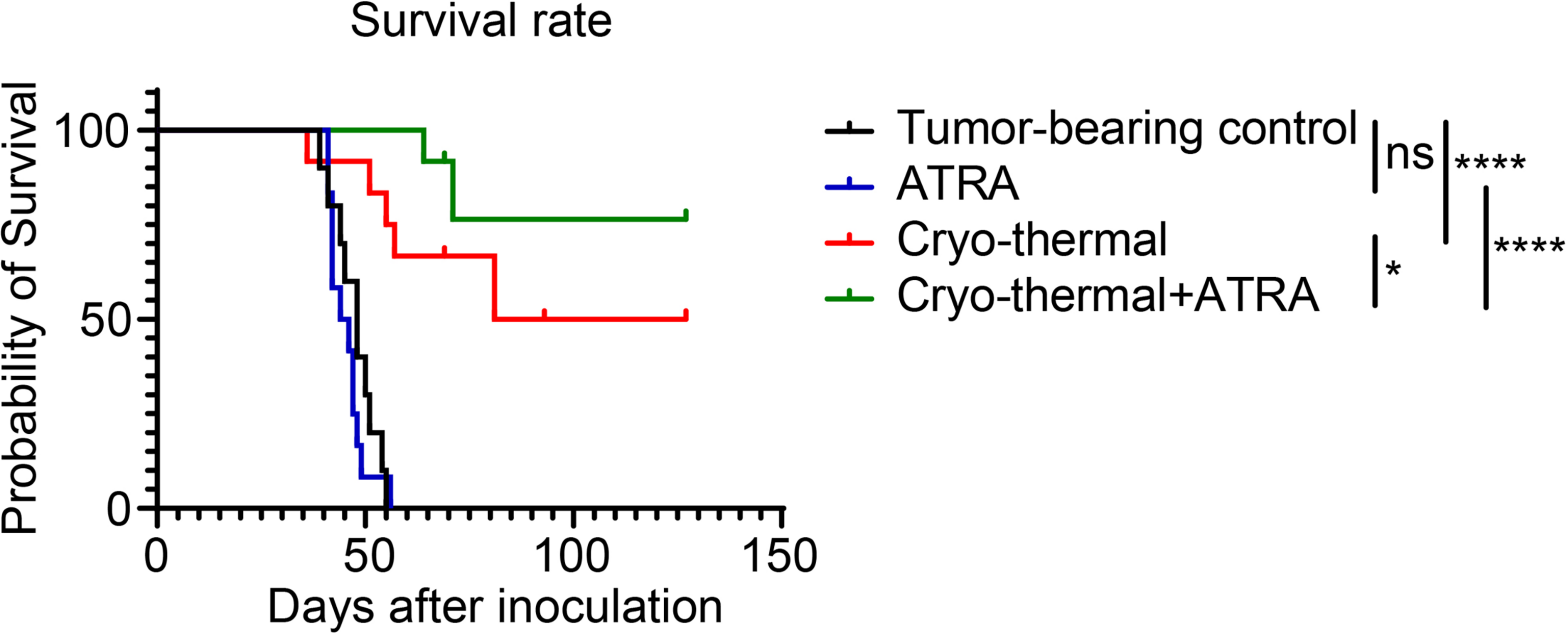
Combination therapy prolonged the long-term survival of 4T1-bearing mice. Kaplan–Meier survival curve of tumor-bearing control, single treatment of ATRA or cryo-thermal therapy, or the combination therapy. The survival curves were compared using log-rank tests. *p < 0.05, ****p < 0.0001. ns, not significant. n = 12 for each group. ATRA, all-trans retinoid acid.

The above results indicated that the combination of ATRA and cryo-thermal therapy could decrease the suppressive function of MDSCs, which inhibited the differentiation of suppressive CD4^+^ T-cell subsets at an early stage and further improved the Th1-dominant differentiation of CD4^+^ T cells at a late stage. To further demonstrate the therapeutic effect of combination therapy, the survival rate was analyzed after the different treatments. As shown in **Figure 6**, the single ATRA treatment did not improve the survival time of mice compared to that of the tumor-bearing control. The long-term survival rate of mice was approximately 50% after cryo-thermal therapy, but after combination therapy, more than 80% of mice survived for a long-term period. These results further suggested that the combination of cryo-thermal therapy and ATRA could obtain a much better therapeutic effect than cryo-thermal therapy alone.

## Discussion

In our previous study, cryo-thermal therapy was reported to induce Th1-dominant CD4^+^ T-cell-mediated antitumor immunity and improve the long-term survival rates of tumor-bearing mice. However, the therapeutic effect in the 4T1 triple-negative breast cancer model was lower than that in the B16F10 melanoma model. We hypothesized that the high level of MDSCs in the 4T1 triple-negative breast cancer model was responsible for the unsatisfactory poor prognosis. To improve the long-term survival rate in the 4T1 triple-negative breast cancer model, in this study, ATRA was used in combination with the cryo-thermal therapy in the 4T1 model. Combination therapy promoted the maturation of MDSCs and reduced their suppressive function by downregulating the inhibitory cytokine pathways and the expression of immune checkpoint molecules, as well as modulating metabolism, which significantly decreased the differentiation of immunosuppressive CD4^+^ T subsets and further facilitated the Th1-dominant differentiation of CD4^+^ T cells to improve the long-term survival rate.

In breast cancer patients, MDSCs are enriched in peripheral circulation, which predicts advanced clinical stage, worse survival, and metastatic extension (24). Moreover, MDSCs participate in the self-seeding of circulating tumor cells leading to the local recurrence in breast cancer patients who received surgery and radiotherapy (25), and MDSCs contribute to the immunosuppressive tumor microenvironment and hinder the therapeutic efficacy. In our previous study, cryo-thermal therapy was administered to a 4T1 model, and approximately half of the mice were cured by cryo-thermal therapy; however, the other mice ultimately died due to tumor metastasis. Moreover, mice with tumor metastasis at the late phase always had higher levels of MDSCs than the mice that survived for a long time (data not shown). Cryo-thermal therapy reduced the proportion of MDSCs and stimulated their maturation (13); however, the level of MDSCs in the 4T1 breast cancer model after cryo-thermal therapy was still higher than that of other tumor models because of the high level of MDSCs in 4T1 model before treatment. In this study, we found that the suppressive function of MDSCs was increased, which mainly hindered Th1-dominant differentiation of CD4^+^ T cells after cryo-thermal therapy alone. Increasing evidence has shown that CD4^+^ T cells, especially the IFN-γ-Th1 subset, are the orchestrator of the antitumor immune response (26). In our previous study, the CD4^+^ Th1 subset increased the cytotoxicity of NK cells and CD8^+^ T cells and promoted the maturation of APCs and MDSCs, resulting in strong and durable antitumor immunity (17). Therefore, although the proportion of the Th1 subset was significantly increased after cryo-thermal therapy, which could be attributed to the decreased proportion of MDSCs, the increased suppressive function of MDSCs could still impair the cryo-thermal-induced antitumor immunity. Our findings indicated that the high suppressive function of MDSCs was responsible for the unsatisfactory therapeutic effects of cryo-thermal therapy in the 4T1 model, and combining cryo-thermal therapy with MDSCs targeting would efficiently improve the long-term survival rate in a high immunosuppressive tumor model.

A number of studies in mouse tumor models have reported that MDSC depletion promotes T cell-dependent antitumor responses (27, 28). In our previous studies, we found that the release of HSP70 induced by cryo-thermal therapy promoted the differentiation of MDSCs into DCs and macrophages (13). Moreover, cryo-thermal-induced M1 macrophages in early-stage therapy increased the differentiation of CD4^+^ Th1 subsets, resulting in the formation of long-term antitumor immune protection to inhibit tumor metastasis (10). Thus, directly depleting MDSCs at an early stage after cryo-thermal therapy could attenuate the expansion of inflammatory DCs and macrophages. Suppressive MDSCs have been reported to be converted into mature myeloid cells that are characterized by the expression of MHC-II or CD86 (29, 30). These mature MDSCs increase expression levels of proinflammatory cytokines and costimulatory molecules and decrease suppressive activity (30). Moreover, the expression of F4/80, CD11c, and MHC-II on MDSCs indicates that the differentiation of MDSCs into potent antigen-presenting cells has a strong capacity to promote Th1 differentiation, as well as enhance the cytotoxic function of CD8^+^ T cells, which leads to a decrease in tumor burden in a Lewis lung carcinoma (LLC) mouse lung cancer model (31). These suggested that promoting the maturation of MDSCs and reprograming their immunosuppressive function may amplify the antitumor immune response induced by cryo-thermal therapy. ATRA was first used in the treatment of acute myeloid leukemia due to its capacity to stimulate the differentiation of MDSCs into DCs and downregulate the inhibitory function of MDSCs (32). However, a single treatment of ATRA fails to prevent tumor development in clinical breast cancer (33). Recently, the combination of ATRA and other immunotherapies, such as PD-1 blockade (34), antiangiogenic therapies (35), and CAR-T (36), significantly inhibit tumor growth and promote long-term survival in a variety of solid tumor models. In this study, a combination of ATRA treatment and cryo-thermal therapy overcame the resistance of MDSC-induced immunosuppression in breast cancer models by promoting the phenotype and functional maturation of MDSCs leading to improve survival rates. Moreover, cryo-thermal therapy has been used in liver metastasis patients and achieved better therapeutic effects (37). Therefore, with the expansion of the indications of cryo-thermal therapy, we suggested that ATRA treatment targeting MDSCs with cryo-thermal therapy would provide a novel therapy for breast cancer in the future.

MDSCs mediate immune suppression through multiple mechanisms, including producing a range of tumorigenic cytokines, especially IL-6, which is a crucial regulator of tumor development and progression (18). Autocrine IL-6 helps to resist necroptosis and facilitate the accumulation of MDSCs by silencing the TNFα-RIP1 cascade (38) and promoting the suppressive capacity of MDSCs by increasing the production of Arg-1, ROS, and PD-L1 while reducing the expression of MHC-II (18). Moreover, the paracrine effect of IL-6 produced by MDSCs can directly increase the survival, proliferation, and drug resistance of tumor cells (39). MDSCs can also express IL-10 and TGF-β, which induce Treg differentiation and promote the suppressive tumor environment. In this study, although the proportion of MDSCs was significantly reduced after cryo-thermal therapy alone, the expression levels of IL-6, IL-10, and TGF-β were also upregulated, which restrain the acute immune response and impaired Th1-dominant CD4^+^ T-cell differentiation (40). Studies have reported that incomplete radiofrequency ablation can accelerate tumor progression and induce constant inflammation, which stimulates the function of myeloid cells (41). In our previous study, tumor lung metastases were established in a 4T1 model before cryo-thermal therapy (13, 14). Therefore, in highly immunosuppressive tumor models, cryo-thermal triggered antitumor immunity may not be strong enough to completely remodel the suppressive tumor environment, resulting in enhancement of the immunosuppressive function of MDSCs at the late stage of treatment. Although the proportion of MDSCs was similar to that after cryo-thermal therapy alone, the expression level of suppressive cytokines in MDSCs was downregulated after combination therapy, which directly inhibited the differentiation of Th2 cells and Tregs at the early stage, leading to Th1-dominant differentiation and stronger cytotoxicity of CD4^+^ T cells at the late stage. A high level of Th1 subsets at the late stage after treatment is characteristic of long-term antitumor immunity (7). In our previous study, we found that Th1 subsets could promote the cytotoxicity of CD8^+^ T and NK cells to increase tumor cell killing (17). Consistently, combination therapy significantly increased the long-term survival rates of mice compared to cryo-thermal therapy alone.

Currently, an increasing number of studies have focused on the clinical therapeutic effects of ATRA and have reported its role in downregulating the proportions of MDSCs and promoting their maturation; however, no evidence has shown that ATRA can directly reprogram the cytokine expression profiles of MDSCs. The metabolic regulation of MDSCs has emerged as a critical modulator in the suppressive tumor environment (42). ATRA can regulate amino acid and fatty acid metabolism (43). Instead of glycolysis, fatty acid oxidation is the main source of energy in tumor-related MDSCs (44). To increase the uptake of fatty acids, fatty acid transport protein 2 (FATP2) is highly expressed in MDSCs, which boosts arachidonic acid uptake and PGE2 synthesis, leading to the acquisition of immunosuppressive activity by MDSCs (45). PGE2 expressed by MDSCs can also upregulate the expression of PD-L1 on MDSCs and is involved in promoting MDSC-induced differentiation of IL-10-expressing T cells (46). Moreover, the increased rate of fatty acid oxidation leads to the upregulation of the expression levels of inhibitory cytokines in MDSCs, including IL-6 and IL-10, as well as granulocyte colony-stimulating factor (G-CSF) and granulocyte-macrophage colony-stimulating factor (GM-CSF), which further accelerate the expansion of MDSCs (22). Glutamine promotes the activation of immature tumor-related myeloid cells and increases Arg-1 expression (47), which limits the proliferation of T cells through cell cycle arrest (48). Targeting glutamine metabolism triggers apoptosis in MDSCs and helps MDSCs differentiate into inflammatory macrophages (21). Moreover, targeting glutamine metabolism can decrease the expression levels of IL-6, IL-1β, iNOS, and Arg-1 in MDSCs and promote the proliferation of T cells, as well as IFN-γ expression (49). These studies suggest that metabolic reprogramming of MDSCs, especially for fatty acid and glutamine restriction, is essential for reducing their suppressive function and stimulating the accumulation and cytotoxicity of T cells. After cryo-thermal therapy, the fatty acid and glutamine metabolism of MDSCs was upregulated compared to that of tumor-bearing controls, which is consistent with the increased expression levels of suppressive cytokines in MDSCs and the promotion of the differentiation of CD4^+^ T immunosuppressive subsets. However, at the late stage after combination therapy, fatty acid and glutamine metabolism were significantly downregulated, and IL-6, IL-10, and TGF-β signaling in MDSCs were reduced. These results suggest that combination therapy mainly reprograms MDSC metabolism and downregulates the expression of suppressive molecules. However, we did not detect the expression level of these differential genes at the protein level, which was a defect.

In conclusion, our study indicated that the combination of ATRA and cryo-thermal therapy could remodel the metabolism of MDSCs and decrease the expression of suppressive molecules, leading to reduce differentiation of immunosuppressive CD4^+^ T-cell subsets, enhance Th1-dominant antitumor immunity, and facilitate long-term survival of mice. Combination therapy with ATRA could act as a new effective strategy in the treatment of tumor patients with high MDSC-induced immunosuppression.

## Data availability statement

The datasets presented in this study can be found in online repositories. The names of the repository/repositories and accession number(s) can be found below: NCBI under accession ID: PRJNA877562.

## Ethics statement

The animal study was reviewed and approved by Shanghai Jiao Tong University Scientific Ethics Committee.

## Author contributions

Conceptualization: YL and PL. Methodology: YL, PP, SW, JW, PD, ZZ, and JZ. Data curation: YL. Writing—original draft preparation: YL. Writing—review and editing: PL and SW. Project administration: PL, and LXX. Funding acquisition: PL and LXX. All authors contributed to the article and approved the submitted version.

## Funding

This work was supported by the National Key Research and Development Program of China (Grant No. 2020YFA0909003), the National Natural Science Foundation of China (Grant No. 82072085), and the Science and Technology Commission of Shanghai Municipality (Grant No. 19DZ2280300 and No. ZJ2021-ZD-007).

## Acknowledgments

Thanks to Dr. Aili Zhang and Engr. Jincheng Zou for manufacturing and maintenance of cryo-thermal therapy system.

## Conflict of interest

The authors declare that the research was conducted in the absence of any commercial or financial relationships that could be construed as a potential conflict of interest.

## Publisher’s note

All claims expressed in this article are solely those of the authors and do not necessarily represent those of their affiliated organizations, or those of the publisher, the editors and the reviewers. Any product that may be evaluated in this article, or claim that may be made by its manufacturer, is not guaranteed or endorsed by the publisher.
