# Supplementary material for: Combining all-trans retinoid acid treatment targeting myeloid-derived suppressive cells with cryo-thermal therapy enhances antitumor immunity in breast cancer
Source: Front Immunol. 2022 Nov 1;13:1016776. doi: 10.3389/fimmu.2022.1016776 (PMC9664198; doi:10.3389/fimmu.2022.1016776)
Supplement: Supplementary file 1 [file DataSheet_1.docx]

Supplementary Material

Supplementary Table 1. Antibodies used in the flow cytometry analysis

| Antibodies | Fluorescence Labeling | Clone | Catalog Number |
| --- | --- | --- | --- |
| CD11b | Pacific Blue | M1/70 | 101224 |
| Gr-1 | PE | RB6-8C5 | 108408 |
| CD86 | APC-cy7 | GL-1 | 105030 |
| MHC-II | Percp-CY5.5 | M5/114.15.2 | 107626 |
| CD11c | FITC | N418 | 117306 |
| F4/80 | APC | BM8 | 123116 |
| CD3 | Percp-CY5.5 | 145-2C11 | 100328 |
| CD4 | APC-cy7 | RM4-5 | 100526 |
| T-bet | PE/Dazzle 594 | 4B10 | 644828 |
| GATA3 | AF488 | 16E10A23 | 653808 |
| Foxp3 | PE | MF-14 | 126404 |
| Bcl-6 | BV421 | K112-91 | 563363 |
| IFN-γ | PE/Dazzle 594 | XMG1.2 | 505846 |
| IL-4 | PE/Dazzle 594 | 11B11 | 504131 |
| IL-17 | APC | TC11-18H10.1 | 506916 |
| Granzyme B | APC | GB11 | 515406 |
| Perforin | PE | S16009A | 154306 |


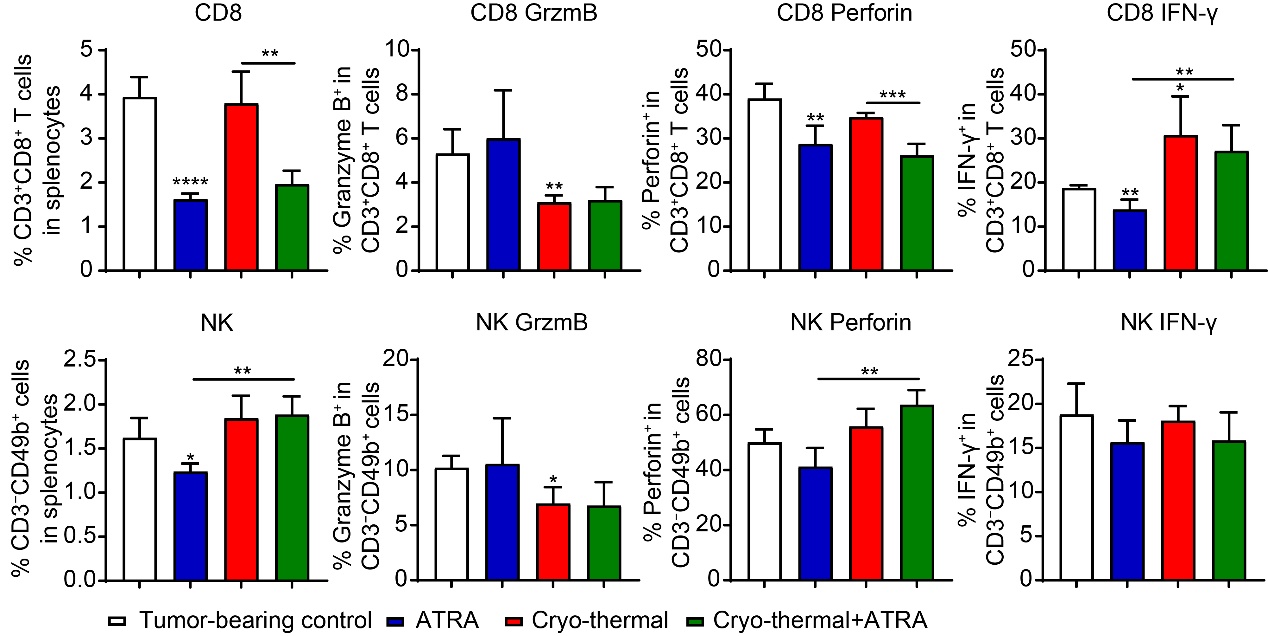


**Supplementary Figure 1.** Combination therapy influenced the proportion and cytotoxicity of CD8^+^ T cells and NK cells at the early stage. *p<0.05, **p<0.01, ***p<0.001, ****p<0.0001. n = 4 for each group.


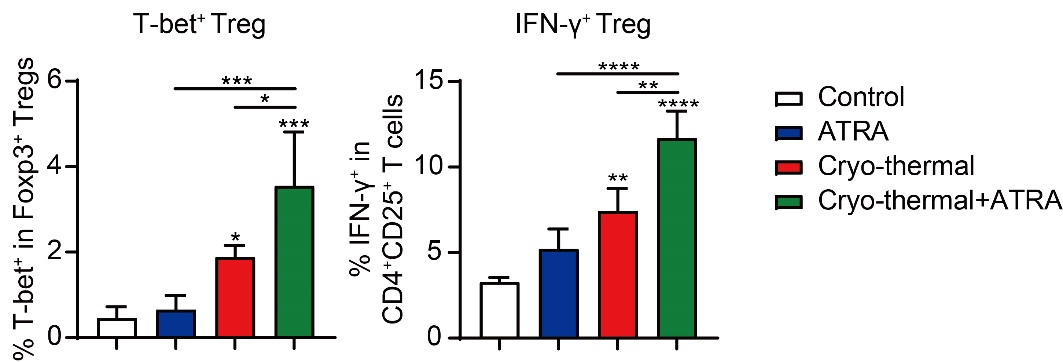


**Supplementary Figure 2.** Combination therapy drove Treg fragility at the late stage. *p<0.05, **p<0.01, ***p<0.001, ****p<0.0001. n = 4 for each group.


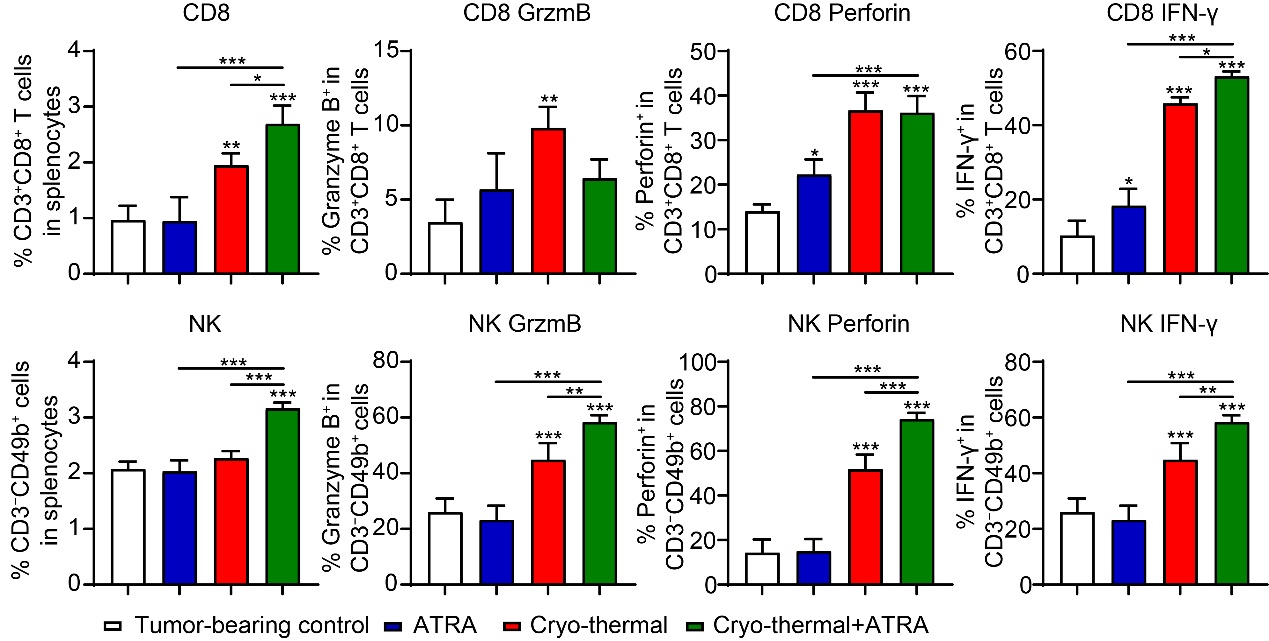


**Supplementary Figure 3.** Combination therapy increased the proportion and cytotoxicity of CD8^+^ T cells and NK cells at the late stage. *p<0.05, **p<0.01, ***p<0.001, ****p<0.0001. n = 4 for each group.


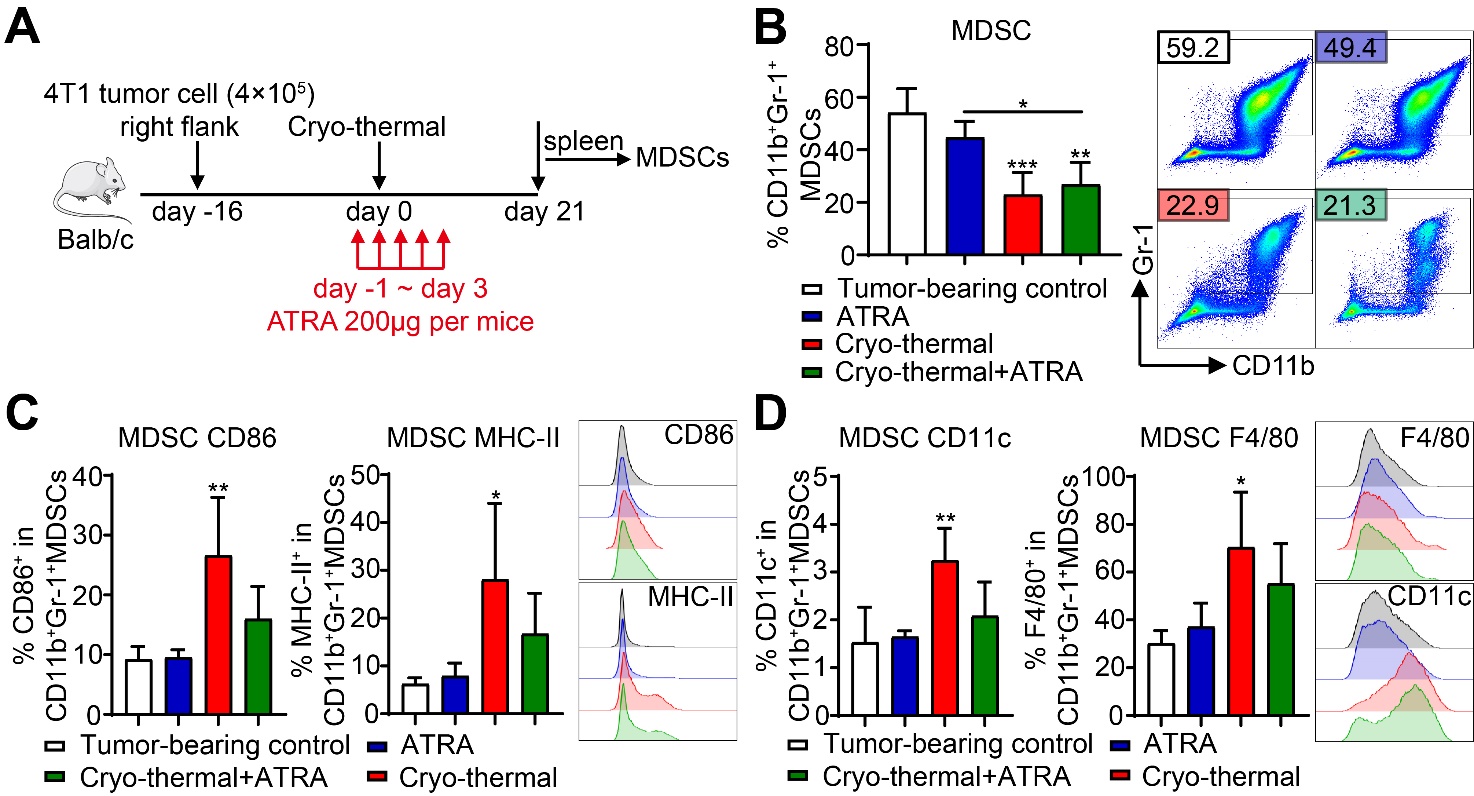


**Supplementary Figure 4.** Combination of cryo-thermal therapy and ATRA facilitate Th1-dominant CD4^+^ T cells differentiation at the late stage. **(A)** Scheme of study design. In brief, 200 μg of ATRA was administered daily from day -1 to 3, the phenotype of MDSCs and the subsets of CD4^+^ T cells were detected on day 21 after cryo-thermal therapy by flow cytometry. **(B-D)** The proportion **(B)**, maturation phenotype **(C)** and transmission phenotype **(D)** of MDSCs. *p<0.05, **p<0.01, ***p<0.001, ****p<0.0001. n = 4 for each group.
